# On Ergodic and Inverse Shadowing Properties of Set-Valued Mapping

**DOI:** 10.12688/f1000research.173380.1

**Published:** 2026-02-20

**Authors:** Farah Wattan Kamil, Iftichar M.T. AL-Shara'a

**Affiliations:** 1 Department of Mathematics, College of Education for Pure Sciences, University of Babylon, Babil, Iraq

**Keywords:** set–valued mappings; shift mapping σ_H; inverse shadowing property.

## Abstract

**Background:**

Shadowing-type properties play a fundamental role in the qualitative theory of dynamical systems, as they describe the relationship between approximate trajectories and exact orbits. In recent years, increasing attention has been given to extending these concepts to set-valued mappings, which naturally arise in various areas of mathematics and applied sciences. However, several shadowing-related notions for such mappings remain insufficiently explored.

**Methods:**

In this work, we introduce precise definitions of the inverse shadowing property and the ergodic shadowing property for set-valued mappings. We analyse these properties within a general topological framework and examine their behaviour under the shift mapping on the inverse limit space. The relationships between inverse shadowing and ergodic shadowing are investigated using tools from topological dynamics.

**Results:**

We establish connections between the inverse shadowing property and the ergodic shadowing property for set-valued mappings. In particular, we show how these properties interact when considered together with the shift mapping on the inverse limit space, and we identify conditions under which one property implies the other.

**Conclusions:**

The results provide a clearer understanding of shadowing phenomena for set-valued mappings and highlight the role of inverse limit spaces in studying their dynamical behavior. This work contributes to the development of shadowing theory beyond single-valued dynamics and offers a foundation for further investigations in this direction.

## 1. Introduction

The phenomenon of shadowing approximation trajectories (pseudo trajectories) of dynamical systems by exact trajectories is one of the most extensively examined issues in contemporary global dynamical systems theory (for example, ^1, 2^). On the other hand, there has begun an equally intense development at the inverse shadowing problem, where a class of methods generating pseudo trajectories is specified and the question is studied as to if it is possible to approximate any precise trajectory by a trajectory of any method from this class (see ^3, 4^).

Román-Flores, ^5^ in 2003, researched the relationship of transitivity with the associated system. After his research, numerous scientists examined the characteristics of set-valued discrete systems. For instance, the transitivity and mixing. ^6, 7^ The ergodic shadowing property is shown by Shabani and Ahmadi ^8^ to be a feature of chain mixing in non-autonomous discrete-time dynamical systems.

Koo and Lee, ^9^ in 2024 proved that the uniform ergodic shadowing characteristic holds for sequences of homeomorphisms on non-compact metric spaces. Al-Sharaa and Russl A., ^10^ in 2023, study generic non-autonomous discrete dynamical systems’ shadowing and w-expansive properties and the relationship between them. In the recent work, ^11^ in 2025, Koo and Lee introduced the notions of dynamical systems on non-compact metric spaces and their ergodic shadowing property.

In this research, we focus on the inverse shadowing property with the ergodic shadowing property in set-valued systems and its relation with inverse limits. We introduce a new definition of the inverse and ergodic shadowing properties of set-valued mappings. This means we will create a space whose elements are sets. We will structure examples that satisfy the new definition in the future research. In the following section, we introduce some important concepts that we need:

### 1.1 Non–autonomous discrete system

Let Ⲭ be represented by any compact metric space, where d is the symbol for the metric on Ⲭ . Let $H_{\eta }:Ⲭ⟶Ⲭ$ , η∈ℕ be a series of mappings represented by $H_{1,\infty }=\left(H_{1},H_{2},\ldots \right)$ . The above sequence indicates a non–autonomous discrete system $\left(Ⲭ,H_{1,\infty }\right)$ . According to this mapping sequence. The point S∈Ⲭ ’s trajectory can be defined as $\mathrm{orb}\left(S,H_{1,\infty }\right)=\left({H_{1}}^{\eta }\left(S\right)\right),\;\eta \in \mathbb{N},$ where ${H_{1}}^{\eta }=H_{\eta }\circ \ldots \circ H_{1}$ , and ${H_{1}}^{0}$ represents the identity mapping similarly ${H_{\eta }}^{k}=H_{\eta +k-1}\circ \ldots \circ H_{\eta +1}\circ H_{\eta }$ .

K(Ⲭ) is the hyperspace of Ⲭ . The space of compact nonempty subsets of Ⲭ where the Hausdorff metric $d_{H}$ is defined as follows: $d_{H}\left(Α,Β\right)=\max\left\{\sup_{S\in Α}\inf_{T\in Β\;}d\left(S,T\right),\sup_{T\in Β}\inf_{S\in Α\;}d\left(S,T\right)\right\}$ for any Α,Β∈K(Ⲭ) . $\left(K\left(Ⲭ\right),d_{H}\right)$ is a compact metric space and H:K(Ⲭ)→K(Ⲭ) is a set–valued mapping on Ⲭ . We call the pair (Ⲭ,H) a set–valued system.

The product topology is applied to ${Ⲭ}^{Ζ}$ , which is the compact metric space of all bi–infinite sequences $ઇ=\left\{S_{k}:k\in \mathbb{Z}\right\}$ in Ⲭ . Let H:K(Ⲭ)→K(Ⲭ) be a set–valued mapping for a constant value δ>0 , where $\Phi_{H}\left(\delta \right)$ represents the set of all δ▁ pseudo trajectories of H . We will provide definitions of inverse shadowing property and ergodic shadowing property for set–valued mapping.

A mapping $ⱷ:Ⲭ\to \Phi_{H}\left(\delta \right)\subseteq {Ⲭ}^{Ζ}$ that satisfies ${ⱷ}_{0}\left(S\right)=S,S\in Ⲭ$ is known as a δ▁ method for H . For any positive ε , there is a positive δ such that for every S in Ⲭ , and any δ▁ method $ⱷ:Ⲭ\to {Ⲭ}^{Ζ}$ , there exists T in Ⲭ , such that $d\left(H^{k}\left(S\right),{ⱷ}_{k}\left(T\right)<\varepsilon \right)$ for any k in ℤ , we say that H has inverse shadowing property, denoted as ISP.

Considering a sequence $ઇ={\left\{S_{¡}\right\}}_{¡\in \mathbb{Z}}$ , take

$$\begin{array}{c} D\left(ઇ,H,\delta \right)=\left\{¡\in \mathbb{Z}:d\left(H\left(S_{¡}\right),S_{¡+1}\right)\geq \delta \right\}\\ D_{\eta }\left(ઇ,H,\delta \right)=D\left(ઇ,H,\delta \right)\cap \left\{-\eta ,\ldots ,-2,-1,0,1,2,\ldots ,\eta \right\} \end{array}$$

In *consideration of* a sequence $ઇ={\left\{S_{¡}\right\}}_{¡\in \mathbb{Z}}$ and a point S∈Ⲭ put

$$\begin{array}{c} DS\left(ઇ,S,H,\delta \right)=\left\{¡\in \mathbb{Z}:d\left(H^{¡}\left(S\right),S_{¡}\right)\geq \delta \right\},\\ DS_{\eta }\left(ઇ,S,H,\delta \right)=DS\left(ઇ,S,H,\delta \right)\cap \left\{-\eta ,\ldots ,-2,-1,0,1,2,\ldots ,\eta \right\}. \end{array}$$

Let $ઇ={\left\{S_{¡}\right\}}_{¡\in \mathbb{Z}}$ be defined as an δ▁ ergodic pseudo trajectories for H if the following limit holds:

$$\lim_{\left|\eta \right|\to \infty }\left(\mathrm{card}\left(D_{\eta }\left(ઇ,H,\delta \right)\right)/\eta \right)=0.$$

An δ▁ ergodic pseudo trajectory is considered ϵ▁ ergodic shadowing with a point S∈Ⲭ denoted by ESP if $\lim_{\left|\eta \right|\to \infty }\left(\mathrm{card}\left(DS_{\eta }\left(ઇ,S,H,\delta \right)\right)/\eta \right)=0$ .

We propose that an H▁ inveriant set Λ possesses an ergodic shadowing property when, for every ϵ>0 , there exists a δ>0 that means all δ▁ ergodic pseudo trajectories in Λ can be ergodic shadowed by a single point S∈Ⲭ .

A closed subspace ${Ⲭ}_{H}=\left\{\left(S_{\eta }\right):S\in Ⲭ,H\left(S_{\eta }\right)=S_{\eta +1}\;,\eta \in \mathbb{Z}\right\}$ of ${Ⲭ}^{Ζ}$ in addition to the corresponding shift mapping $\sigma_{H}:{Ⲭ}_{H}\to {Ⲭ}_{H}$ , which is defined as $\sigma_{H}\left((S_{\eta })\right)=\left(T_{\eta }\right)$ where $T_{\eta }=S_{\eta +1}$ for all η∈ℤ is referred to as the inverse limit space of H . It is important to observe that the compact metric space ${Ⲭ}_{H}$ is determined by the metric $\tilde{d}$ , which is defined as $\tilde{d}\left((S_{¡}),(T_{¡})\right)=\sum_{¡\in \mathbb{Z}}\left(d\left(S_{¡},T_{¡}\right)/2^{\left|¡\right|}\right)$ .

For each ¡,j∈ℤ , consider ¡ the projection mapping $\pi_{¡}:{Ⲭ}_{H}\to K\left(Ⲭ\right)$ by $\pi_{¡}\left(S\right)=S_{¡}$ for any $\tilde{S}\in {Ⲭ}_{H}$ where $\tilde{S}=\left\{\ldots ,S_{¡-1},S_{¡},S_{¡+1},\ldots \right\}$ , for any ¡∈ℤ , the mapping $\pi_{i}$ satisfies that $H\circ \pi_{¡}=\pi_{¡}\circ \sigma_{H}$ as it is an open continuous mapping.

## 2. Results

Theorem 2.1. A continuous surjective set–valued mapping on a compact metric space Ⲭ is denoted by H:K(Ⲭ)→K(Ⲭ) . Assuming H has ESP, the corresponding shift mapping $\sigma_{H}$ on the inverse limit space ${Ⲭ}_{H}$ also has ESP.

Proof: Let ϵ be greater than zero; then diam(Ⲭ)=α we can select N from ℕ such that $\left(\frac{\alpha }{2^{N-1}}\right)<\left(\frac{\epsilon }{8}\right)$ . Since d(S,T)<γ , the uniform continuity of H ensures that $d\left(H^{¡}\left(S\right),H^{¡}\left(T\right)\right)<\left(\frac{\epsilon }{8}\right)$ for 0≤¡≤2N . Given that H has ESP. Any τ▁ ergodic pseudo trajectory is γ▁ ergodic shadowed by a point in Ⲭ for any γ>0 there is τ>0 . Get δ>0 so that $0<\delta 2^{N}<\tau$ .

Suppose ${\left\{S^{\eta }\right\}}_{\eta \in \mathbb{Z}}\subseteq {Ⲭ}_{H}$ be a δ▁ ergodic pseudo trajectory with $\sigma_{H}$ . Because $\left\{\eta \backslash d\left(H\left(S_{-N}^{\eta }\right),S_{-N}^{\eta +1}\right)\geq \tau \right\}\subseteq \left\{\eta \backslash \tilde{d}\left(\sigma_{H}\left(S^{\eta }\right),S^{\eta +1}\right)\geq \delta \right\}$ the sequence of $\left\{S_{-N}^{\eta }\right\}$ forms a τ▁ ergodic pseudo trajectory for H . However, there exists T∈Ⲭ that means $\lim_{\left|m\right|\to \infty }\left(\mathrm{card}\left(\left\{\eta \backslash d\left(H^{\eta }\left(T\right),S_{-N}^{\eta }\right)\geq \gamma \right\}\cap \left\{-m+1,\ldots ,m-1\right\}\right)\right)\times m^{-1}=0$ .

Suppose $T_{¡-N}=H^{¡}\left(T\right)$ for any ¡≥0 and $T_{¡-N}\in H^{-1}\left(T_{¡+1-N}\right)$ for ¡<0 , the $\tilde{T}=\left(T_{¡}\right)\in {Ⲭ}_{H}$ . If $\eta \notin \left\{\eta /d\left(H^{\eta }\left(T\right),S^{\eta }\right)\geq \gamma \right\}$ then $\eta \notin \left\{\eta /\tilde{d}\left(\sigma^{\eta }\left(\tilde{T}\right),S^{\eta }\right)\geq \epsilon \right\}$ which is to say $\left\{\eta /\tilde{d}\left(\sigma^{\eta }\left(\tilde{T}\right),S^{\eta }\right)\geq \epsilon \right\}\subseteq \left\{\eta /d\left(H^{\eta }\left(T\right),S_{-N}^{\eta }\right)\geq \gamma \right\}$ . Consequently, $\left\{S^{\eta }\right\}\subseteq {Ⲭ}_{H}$ could display ϵ▁ ergodic shadowed for $\tilde{T}\in {Ⲭ}_{H}$ . ■

Theorem 2.2. A continuous surjective set–valued mapping on a compact metric space Ⲭ is denoted by H:K(Ⲭ)→K(Ⲭ) . Assuming H has ISP, the corresponding shift mapping $\sigma_{H}$ on the inverse limit space ${Ⲭ}_{H}$ also has ISP.

Proof: Let ϵ be greater than zero; then diam(Ⲭ)=α we can select N from ℕ so that $\left(\frac{\alpha }{2^{N-1}}\right)<\left(\frac{\epsilon }{8}\right)$ . Since d(S,T)<γ , the uniform continuity of H ensures that $d\left(H^{¡}\left(S\right),H^{¡}\left(T\right)\right)<\left(\frac{\epsilon }{8}\right)$ for 0≤¡≤2N .

Given that H has ISP, for each γ>0 ,there is τ>0 which means that with each S∈Ⲭ and every τ▁ method ⱷ , it exists T∈Ⲭ satisfying $d\left(H^{k}\left(S\right),{ⱷ}_{k}\left(T\right)\right)<\gamma$ , for all k∈ℤ .

Select δ>0 such that $0<\delta 2^{N}<\tau$ . suppose that $\tilde{ⱷ}:{Ⲭ}_{H}$ → ${\tilde{\Phi }}_{\sigma }$ is a δ▁ method for $\sigma_{H}$ . We construct a τ▁ method of H this way: let S∈Ⲭ , choose a point $\tilde{S}=\left(S_{¡}\right)\in {Ⲭ}_{H}$ where $\pi_{-N}\left(\tilde{S}\right)=S$ . If $\tilde{ⱷ}$ constitutes a δ▁ method of $\sigma_{H}$ , therefore, $\tilde{S}\in {Ⲭ}_{H}$ , $d\left(\sigma_{H}\left({\tilde{ⱷ}}_{\eta }\left(\tilde{S}\right)\right),{\tilde{ⱷ}}_{\eta +1}\left(\tilde{S}\right)\right)<\delta$ .

Let $ⱷ:Ⲭ\to {Ⲭ}^{Ζ}$ be defined as $ⱷ\left(S\right)={\left\{{ⱷ}_{\eta }\left(S\right)\right\}}_{\eta \in \mathbb{Z}}$ , where ${ⱷ}_{\eta }\left(S\right)=\pi_{-N}\left({\tilde{ⱷ}}_{\eta }\left(\tilde{S}\right)\right)$ . It is evident that ⱷ(S) forms a τ▁ pseudo trajectory for H ; hence, ⱷ behaves as a τ▁ method for H . Since H is ISP, every $\pi_{-N}\left(\tilde{S}\right)\in Ⲭ$ , where $\tilde{S}\in {Ⲭ}_{H}$ it has T∈Ⲭ that gives $d\left(H^{\eta }\left(\pi_{-N}\left(\tilde{S}\right)\right),{ⱷ}_{\eta }\left(T\right)\right)<\gamma$ . If $\tilde{T}\in {Ⲭ}_{H}$ where $\pi_{-N}\left(\tilde{T}\right)=T$ ; then $d\left(\sigma_{H}^{\eta }\left(\tilde{S}\right),{\tilde{ⱷ}}_{\eta }\left(\tilde{T}\right)\right)<\epsilon$ ; this means that $\sigma_{H}$ has ISP. ■

Theorem 2.3. A local homeomorphism set–valued mapping on a compact metric space Ⲭ is denoted by H:K(Ⲭ)→K(Ⲭ) . Assuming the shift mapping $\sigma_{H}$ on ${Ⲭ}_{H}$ has ESP, then H also has ESP.

Proof: For each ϵ>0 , according to the ESP of $\sigma_{H}$ , it has a τ>0 for every τ▁ ergodic pseudo trajectory in $\sigma_{H}$ a point in ${Ⲭ}_{H}$ that forms ϵ▁ ergodic shadowed. Let α=diam(Ⲭ) , and choose N∈ℕ so that $\left(\frac{\alpha }{2^{N-1}}\right)<\left(\frac{\tau }{4}\right)$ .

H be the local homeomorphism; it has a γ such that 0<γ<(τ/4) and H|U:U→U forms a homeomorphism when U defines the γ▁ neighborhood of S∈Ⲭ . H being uniformly continuous, for τ/8 , there is 0<Γ<(τ/8) where d(S,T)<Γ means $d\left(H^{j}\left(S\right),H^{j}\left(T\right)\right)<\left(\tau /8\right)$ for every j,|j|≤N .

Let ${\left\{S_{\eta }\right\}}_{\eta \in \mathbb{Z}}$ be a Γ▁ ergodic pseudo trajectory for H ; define $S_{0}^{\eta }=S_{\eta }$ and $S^{\eta }=\left(S_{¡}^{\eta }\;\right)\in {Ⲭ}_{H}$ . If $\eta \notin \left\{\eta \;|\;d\left(H\left(S_{\eta }\right),S_{\eta +1}\right)\geq \Gamma \right\}$ , then $\eta \notin \left\{\eta |\;\tilde{d}\left(\sigma_{H}\left(S^{\eta }\right),S^{\eta +1}\right)\geq \tau \right\}$ . This shows that $\left\{\eta |\;\tilde{d}\left(\sigma_{H}\left(S^{\eta }\right),S^{\eta +1}\right)\geq \tau \right\}\subseteq \left\{\eta \;|\;d\left(H\left(S_{\eta }\right),S_{\eta +1}\right)\geq \Gamma \right\}$ , and hence $\left\{S^{\eta }\right\}$ defines a τ▁ ergodic pseudo trajectory for $\sigma_{H}$ . Since $\sigma_{H}$ has ESP, there is $\tilde{T}=\left(T_{i}\right)$ in ${Ⲭ}_{H}$ such that $\lim_{|m|\;\to \;\infty }\left(\left\{\eta \;|\;\tilde{d}\left({\sigma_{H}}^{\eta }\left(\;\tilde{T}\right),S^{\eta }\right)\geq \epsilon \right\}⋂\left\{-m+1,\ldots ,m-1\right\}/m\right)=0$ .

If $\eta \notin \left\{\eta \;|\;\tilde{d}\left({\sigma_{H}}^{\eta }\left(\tilde{T}\right),S^{\eta }\right)\geq \epsilon \right\}$ , then $\eta \notin \left\{\eta \;|\;d\left(H^{\eta }\left(T_{0}\right),S_{0}^{\eta }\right)\geq \epsilon \right\}$ , and we get $\left\{\eta \;|\;d\left(H^{\eta }\left(T_{0}\right),S_{\eta }\right)\geq \epsilon \right\},\subseteq \left\{\eta \;|\;\tilde{d}\left({\sigma_{H}}^{\eta }\left(T\right),S^{\eta }\right)\geq \epsilon \right\}$ . It is now evident by definition that H has ESP.■

Theorem 2.4. A local homeomorphism set–valued mapping on a compact metric space Ⲭ is denoted by H:K(Ⲭ)→K(Ⲭ) . Assuming the shift mapping $\sigma_{H}$ on ${Ⲭ}_{H}$ has ISP, then H also has ISP.

Proof: For each ϵ>0 by ISP of $\sigma_{H}$ , there is τ>0 such that, for each of $\tilde{S}\in {Ⲭ}_{H}$ and for every τ▁ method $\tilde{ⱷ}$ of $\sigma_{H}$ , there is $\tilde{T}\in {Ⲭ}_{H}$ so that $\tilde{d}\left({\sigma_{H}}^{\eta }\left(\tilde{S}\right),{\tilde{ⱷ}}_{\eta }\left(\tilde{T}\right)\right)<\epsilon$ .

Let α=diam(Ⲭ) , select N∈ℕ so that $\left(\frac{\alpha }{2^{N-1}}\right)<\left(\frac{\tau }{4}\right)$ . H be a local homeomorphism; U represents the γ▁ neighborhood of S∈Ⲭ , and H|U:U→U is a homeomorphism if there is a γ such that 0<γ<(τ/4) . Since H represents uniform *continuity* at τ/8 ; for every j where |j|≤N there exists 0<Γ<(τ/8) such that d(S,T)<Γ and $d\left(H^{j}\left(S\right),H^{j}\left(T\right)\right)<\left(\tau /8\right)$ .

Let S be an element of Ⲭ , and define $ⱷ:Ⲭ\to \Phi_{H}\left(\delta \right)$ as a γ▁ method of H . Let $\tilde{ⱷ}:{Ⲭ}_{H}$ → ${{Ⲭ}_{H}}^{\mathbb{Z}}$ so that $\tilde{ⱷ}\left(\tilde{S}\right)={\left\{{\tilde{ⱷ}}_{\eta }\left(\tilde{S}\right)\right\}}_{\eta \in \mathbb{Z}}$ with $\pi_{0}\left(\tilde{ⱷ}\left(\tilde{S}\right)\right)={ⱷ}_{\eta }\left(S\right)$ we have $\tilde{d}\left(\sigma_{H}\left({\tilde{ⱷ}}_{\eta }\left(\tilde{S}\right)\right),{\tilde{ⱷ}}_{\eta +1}\left(\tilde{S}\right)\right)<\delta$ . Therefore $\tilde{ⱷ}:{Ⲭ}_{H}$ → ${{Ⲭ}_{H}}^{\mathbb{Z}}$ represents a δ▁ method for $\sigma_{H}$ , since $\sigma_{H}$ has the ISP; thus, given $\tilde{S}\in {Ⲭ}_{H}$ there is $\tilde{T}\in {Ⲭ}_{H}$ , which means $\tilde{d}\left({\sigma_{H}}^{\eta }\left(\tilde{S}\right),{\tilde{ⱷ}}_{\eta }\left(\tilde{T}\right)\right)<\epsilon$ , this implies that $d\left(H^{\eta }\left(S\right),{ⱷ}_{\eta }\left(T\right)\right)<\epsilon$ ; hence, H has the ISP. ■

## 3. Conclusion

In this paper, we present a new definition of the inverse and ergodic shadowing properties for set-valued mappings. We explore the relationship between the inverse shadowing property and the ergodic shadowing property of set-valued mappings, as well as their connection to the shift mapping $\sigma_{H}$ on the inverse limit space.

## Data Availability

Our article type does not require data.
