# On Ergodic and Inverse Shadowing Properties of Set-Valued Mapping

**DOI:** 10.12688/f1000research.173380.2

**Published:** 2026-04-16

**Authors:** Farah Wattan Kamil, Iftichar M.T. AL-Shara'a

**Affiliations:** 1 Department of Mathematics, College of Education for Pure Sciences, University of Babylon, Babil, Iraq

**Keywords:** set–valued mappings; shift mapping σ_H; inverse shadowing property.

## Abstract

This paper examines the correlation between the inverse shadowing property and the ergodic shadowing property for set-valued mappings. First, we present appropriate definitions of inverse shadowing and ergodic shadowing properties within the context of set-valued dynamical systems. We then examine these properties through the shift mapping σ_H on the inverse limit space related to the mapping. We derive several results elucidating the behavior of these shadowing properties under the induced dynamical system on the inverse limit space. We specifically delineate the conditions necessary for the preservation of the ergodic shadowing property in the shift mapping and examine its relationship with the inverse shadowing property in set-valued mappings. These findings broaden established correlations between shadowing properties and inverse limit spaces from single-valued dynamical systems to the field of set-valued dynamical systems.

## 1. Introduction

The shadowing property, which describes the approximation of pseudo-trajectories by exact trajectories in dynamical systems, has been widely studied in modern global dynamical systems theory (for example, ^1, 2^). This concept plays an important role in understanding the stability of dynamical behavior, since it guarantees that approximate trajectories produced by numerical computations or perturbations can be closely followed by true trajectories of the system.

In contrast to the classical shadowing problem, the inverse shadowing problem investigates whether every exact trajectory of a dynamical system can be approximated by trajectories generated by certain methods that produce pseudo-trajectories (see ^3^ ^,^ ^4^). The study of inverse shadowing provides another perspective for analyzing the robustness of dynamical systems and has attracted increasing attention in recent years.

Set-valued dynamical systems have also received considerable interest in the literature. In this direction, Román-Flores, ^5^ studied the relationship between transitivity and the associated dynamical system. Following this work, several authors investigated dynamical properties of set-valued discrete systems, including transitivity and mixing properties. ^6^ ^,^ ^7^ Furthermore, Shabani and Ahmadi ^8^ showed that the ergodic shadowing property is related to chain mixing in non-autonomous discrete-time dynamical systems.

More recently, further developments have appeared in the study of ergodic shadowing. In particular, Koo and Lee, ^9^ proved that the uniform ergodic shadowing property holds for sequences of homeomorphisms on non-compact metric spaces. In another work, Al-Sharaa and Russl A., ^10^ investigated the shadowing and w -expansive properties for generic non-autonomous discrete dynamical systems and analyzed the relationship between them. More recently, Koo and Lee, ^11^ introduced dynamical systems on non-compact metric spaces and studied their ergodic shadowing property.

Motivated by these developments, in this paper we study the relationship between the inverse shadowing property and the ergodic shadowing property for set-valued mappings, and analyze these properties through the associated inverse limit space. We introduce suitable definitions of inverse shadowing and ergodic shadowing properties for set-valued mappings and investigate how these properties behave for the induced dynamical system on the inverse limit space. Our results extend several known relationships between shadowing properties and inverse limit spaces from single-valued dynamical systems to the framework of set-valued dynamical systems.

The remainder of the paper is organized as follows. In the next section, we present several basic definitions and preliminary concepts that will be used throughout the paper.

## 2. Preliminaries

Let X be a compact metric space with metric d . Let ${\left\{H_{\eta }\right\}}_{\eta \in \mathbb{N}}$ be a sequence of mappings $H_{\eta }:X⟶X$ . Then the sequence $H_{1,\infty }=\left(H_{1},H_{2},\ldots \right)$ defines a non–autonomous discrete system $\left(X,H_{1,\infty }\right)$ . For any point x∈X , its trajectory is defined by $\mathrm{orb}\left(x,H_{1,\infty }\right)=\left({H_{1}}^{\eta }\left(x\right):\eta \in \mathbb{N}\;\right)$ , where ${H_{1}}^{\eta }=H_{\eta }\circ \ldots \circ H_{1}$ , and ${H_{1}}^{0}$ denotes the identity mapping similarly ${H_{\eta }}^{k}=H_{\eta +k-1}\circ \ldots \circ H_{\eta +1}\circ H_{\eta }$ .

Let K(X) denote the hyperspace of all non-empty compact subsets of X , endowed with the Hausdorff metric $d_{H}$ , defined by $d_{H}\left(Α,Β\right)=\max\left\{\sup_{x\in Α}\inf_{y\in Β\;}d\left(x,y\right),\sup_{y\in Β}\inf_{x\in Α\;}d\left(x,y\right)\right\}$ for any Α,Β∈K(X) . Then $\left(K\left(X\right),d_{H}\right)$ is a compact metric space.

Let H:X→K(X) be a set–valued mapping, that is, for each x∈X , the image H(x) is a non-empty compact subset of X . The pair (X,H) is called a set–valued dynamical system.

We denote by $X^{Ζ}$ the set of all bi–infinite sequences in X , that is, $X^{Ζ}=\left\{{\left(x_{¡}\right)}_{¡\in \mathbb{Z}}:x_{¡}\in X\right\}$ . This space is endowed with the product topology, under which it is a compact metric space.

A sequence ${\left\{x_{¡}\right\}}_{¡\in \mathbb{Z}}\;\subset \;X$ is called a trajectory of H if $x_{¡+1}\in H\left(x_{¡}\right)$ , for all ¡∈ℤ . Let δ>0 . A sequence ${\left\{x_{¡}\right\}}_{¡\in \mathbb{Z}}\;\subset \;X$ is called a δ▁ pseudo trajectory of H if $d\left(H\left(x_{¡}\right),x_{¡+1}\;\right)<\delta$ , for all ¡∈ℤ , where the distance between a set and a point is defined by $d\left(H\left(x_{¡}\right),x_{¡+1}\;\right)=\underset{y\in H\left(x_{¡}\right)}{\inf}d\left(y,x_{¡+1}\;\right)$ .

Let $\Phi_{H}\left(\delta \right)$ denote the set of all δ▁ pseudo trajectories of H . A mapping $ⱷ:X\to X^{Ζ}$ satisfying ${ⱷ}_{0}\left(x\right)=x$ , for all x∈X is called a δ▁ method for H .

We say that H has inverse shadowing property (ISP) if for every ϵ>0 , there exists δ>0 such that for every δ▁ method ⱷ and every x∈X , there exists y∈X such that $d\left(x_{¡},{ⱷ}_{¡}\left(y\right)\right)<\epsilon$ , for all ¡∈ℤ , where ${\left\{x_{¡}\right\}}_{¡\in \mathbb{Z}}$ is a trajectory of H with $x_{0}=x$ .

For a sequence ${\left\{x_{¡}\right\}}_{¡\in \mathbb{Z}}$ , define

$$D\left(\{x_{¡}\},H,\delta \right)=\left\{¡\in \mathbb{Z}:d\left(H\left(x_{¡}\right),x_{¡+1}\right)\geq \delta \right\},$$

and

$$D_{\eta }\left(\{x_{¡}\},H,\delta \right)=D\left(\{x_{¡}\},H,\delta \right)\cap \left[-\eta ,\eta \right].$$

The sequence ${\left\{x_{¡}\right\}}_{¡\in \mathbb{Z}}$ is called a δ▁ ergodic pseudo trajectory if

$$\lim_{\eta \to \infty }\left(\mathrm{card}\left(D_{\eta }\left(\{x_{¡}\},H,\delta \right)\right)/2\eta +1\right)=0.$$

Let x∈X . Define

$$DS\left(\{x_{¡}\},x,H,\delta \right)=\left\{¡\in \mathbb{Z}:d\left(x_{¡},\overset{´}{x_{¡}}\right)\geq \delta \right\},$$

Where $\left\{\overset{´}{x_{¡}}\right\}$ is a trajectory of H with ${\overset{´}{x}}_{0}=x$ and

$$DS_{\eta }\left(\{x_{¡}\},x,H,\delta \right)=DS\left(\{x_{¡}\},x,H,\delta \right)\cap \left[-\eta ,\eta \right].$$

We say that $\left\{x_{¡}\right\}$ is

ϵ▁

ergodically shadowed by x if

$$\lim_{\eta \to \infty }\left(\frac{\mathrm{card}\left(DS_{\eta }\left(\{x_{¡}\},x,H,\epsilon \right)\right)}{2\eta +1}\right)=0.$$

We say that H has the ergodic shadowing property (ESP) if every ϵ>0 , there exists a δ>0 such that every δ▁ ergodic pseudo trajectory is ϵ▁ ergodically shadowed by some point in X .

The inverse limit space associated with H is defined by $X_{H}=\left\{{\left\{x_{¡}\right\}}_{¡\in \mathbb{Z}}\in X^{Ζ}:x_{¡+1}\in H\left(x_{¡}\right)\right\}$ . The shift mapping $\sigma_{H}:X_{H}\to X_{H}$ is defined by $\sigma_{H}\left((x_{¡})\right)=\left(x_{¡+1}\right)$ . The space $X_{H}$ is endowed with the metric $\tilde{d}\left((x_{¡}),(y_{¡})\right)=\sum_{¡\in \mathbb{Z}}\left(d\left(x_{¡},y_{¡}\right)/2^{\left|¡\right|}\right)$ .

For each ¡∈ℤ , define the projection mapping $\pi_{¡}:X_{H}\to X$ by $\pi_{¡}\left({\left\{x_{j}\right\}}_{j\in \mathbb{Z}}\right)=x_{¡}$ for any ${\left\{x_{j}\right\}}_{j\in \mathbb{Z}}\in X_{H}$ . Then for every ¡∈ℤ , the mapping $\pi_{¡}$ is continuous. Moreover, it satisfies $\pi_{¡}\;\circ \;\sigma_{H}=\pi_{¡+1}$ .

In addition, by the definition of the inverse limit space, we have $\pi_{¡+1}\left(x\right)\in H\left(\pi_{¡}\left(x\right)\right)$ for all $x\in X_{H}$ .

## 3. Results

Theorem 3.1. Let X be a compact metric space and let H be a continuous and surjective set–valued mapping. If H has ESP, then the shift mapping $\sigma_{H}$ on the inverse limit space $X_{H}$ also has ESP.

Proof: Let ϵ>0 . Since X is compact, let α=diam(X) . Choose N∈ℕ such that $\left(\frac{\alpha }{2^{N-1}}\right)<\left(\frac{\epsilon }{8}\right)$ . By the uniform continuity of H , there exists γ>0 such that $d\left(x,y\right)<\gamma ⟹d\left(H\left(x\right),H\left(y\right)\right)<\left(\frac{\epsilon }{8}\right)$ . By induction, this implies that $d\left(x,y\right)<\gamma ⟹d\left(H^{¡}\left(x\right),H^{¡}\left(y\right)\right)<\left(\frac{\epsilon }{8}\right)$ for 0≤¡≤2N .

Since H has ESP, for this γ , there exists τ>0 such that every τ▁ ergodic pseudo trajectory of H is γ▁ ergodically shadowed by some point in X .

Now choose δ>0 so that $0<\delta 2^{N}<\tau$ .

Let ${\left\{S^{k}\right\}}_{k\in \mathbb{Z}}\subseteq X_{H}$ be a δ▁ ergodic pseudo trajectory of $\sigma_{H}$ , where $S^{k}={\left\{{x_{¡}}^{k}\right\}}_{¡\in \mathbb{Z}}$ .

Then by definition of the shift mapping and the metric $\tilde{d}$ , we have $\tilde{d}\left(\sigma_{H}\left(S^{k}\right),S^{k+1}\right)=\sum_{¡\in \mathbb{Z}}\left(d\left(x_{¡+1}^{k},x_{¡}^{k+1}\right)/2^{\left|¡\right|}\right)$ . From this and the condition $\tilde{d}\left(\sigma_{H}\left(S^{k}\right),S^{k+1}\right)<\delta$ , it follows that $d\left(x_{-N+1}^{k},x_{-N}^{k+1}\right)<\delta 2^{N}$ , thus $d\left(x_{-N+1}^{k},x_{-N}^{k+1}\right)<\tau$ . Hence, the sequence ${\left\{x_{-N}^{k}\right\}}_{k\in \mathbb{Z}}$ is a τ▁ ergodic pseudo trajectory of H .

So that, there exists a point y∈X such that ${\left\{x_{-N}^{k}\right\}}_{k\in \mathbb{Z}}$ is γ▁ ergodically shadowed by the trajectory of y , i.e.,

$$\lim_{\eta \to \infty }\frac{1}{2\eta +1}\left(\mathrm{card}\left(k\in \left[-\eta ,\eta \right]:d\left(x_{-N}^{k},y_{k}\right)\geq \gamma \right)\right)=0$$

Where $y_{k}$ is a trajectory of H . Using the surjectivity of H , we can construct a sequence $\tilde{y}={\left\{y_{¡}\right\}}_{¡\in \mathbb{Z}}\subset \;X_{H}$ such that $y_{¡+1}\in H\left(y_{¡}\right),\forall ¡\in \mathbb{Z}$ . So $\tilde{d}\left({\sigma_{H}}^{k}\left(\tilde{y}\right),S^{k}\right)<\epsilon$ , for all indices except a set of density zero.

Hence, $\left\{S^{k}\right\}$ is ϵ▁ ergodically shadowed by $\tilde{y}$ . Therefore, the shift mapping $\sigma_{H}$ has the ESP.■

Theorem 3.2. Let X be a compact metric space and H be a continuous and surjective set-valued mapping. If H has ISP, then the shift mapping $\sigma_{H}$ ​ on the inverse limit space $X_{H}$ also has ISP.

Proof: Let ϵ>0 . Since X is compact, let α=diam(X) . Choose N∈ℕ such that $\left(\frac{\alpha }{2^{N-1}}\right)<\left(\frac{\epsilon }{8}\right)$ . By the uniform continuity of H , there exists γ>0 such that $d\left(x,y\right)<\gamma ⟹d\left(H\left(x\right),H\left(y\right)\right)<\left(\frac{\epsilon }{8}\right)$ . By induction, this implies that $d\left(x,y\right)<\gamma ⟹d\left(H^{¡}\left(x\right),H^{¡}\left(y\right)\right)<\left(\frac{\epsilon }{8}\right)$ for 0≤¡≤2N .

Since H has ISP, for this γ>0 , there exists τ>0 such that for every τ▁ method ⱷ and every x∈X , there exists y∈X such that $d\left(x_{k},{ⱷ}_{k}\left(y\right)\right)<\gamma$ for all k∈ℤ , where $\left\{x_{k}\right\}$ is a trajectory of H .

Now choose δ>0 so that $0<\delta 2^{N}<\tau$ . Let $\tilde{ⱷ}:X_{H}\;\to \;{X_{H}}^{Ζ}$ is a δ▁ method for $\sigma_{H}$ . Take any x∈X . By surjectivity of H , there exists $\tilde{x}=\left(x_{¡}\right)\in X_{H}$ such that ⱷ(x) t $\pi_{-N}\left(\tilde{x}\right)=x$ . Define $={\left\{{ⱷ}_{k}\left(x\right)\right\}}_{k\in \mathbb{Z}}$ , where ${ⱷ}_{k}\left(x\right)=\pi_{-N}\left({\tilde{ⱷ}}_{k}\left(\tilde{x}\right)\right)$ .

Using the definition of $\tilde{d}$ , we have $\tilde{d}\left(\sigma_{H}\left({\tilde{ⱷ}}_{k}\left(\tilde{x}\right)\right),{\tilde{ⱷ}}_{k+1}\left(\tilde{x}\right)\right)<\delta$ . Thus, for coordinate −N : $d\left(x_{-N+1},x_{-N}^{k+1}\right)<\delta 2^{N}<\tau$ . Hence, ⱷ(x) is τ▁ pseudo trajectory for H , and therefore ⱷ is a τ▁ method for H . Since H is ISP, there exists y∈X such that $d\left(x_{k},{ⱷ}_{k}\left(y\right)\right)<\gamma$ for all k∈ℤ . Choose $\tilde{y}\in X_{H}$ such that $\pi_{-N}\left(\tilde{y}\right)=y$ . We get $\tilde{d}\left(\sigma_{H}^{k}\left(\tilde{x}\right),{\tilde{ⱷ}}_{k}\left(\tilde{y}\right)\right)<\epsilon$ . Thus, $\sigma_{H}$ has ISP.■

Theorem 3.3. Let X be a compact metric space and let H be a continuous set-valued mapping which is locally a homeomorphism. If the shift mapping $\sigma_{H}$ ​ on the inverse limit space $X_{H}$ ​ has ESP, then H also has ESP.

Proof: Let ϵ>0 . Since $\sigma_{H}$ has the ESP, there exists τ>0 such that every τ▁ ergodic pseudo trajectory in $X_{H}$ is ϵ▁ ergodically shadowed by some point in $X_{H}$ . Let α=diam(Ⲭ) , and choose N∈ℕ so that $\left(\frac{\alpha }{2^{N-1}}\right)<\left(\frac{\tau }{4}\right)$ .

Since H is uniformly continuous, there exists Γ>0 such that d(x,y)<Γ means $d\left(H^{¡}\left(x\right),H^{¡}\left(y\right)\right)<\left(\tau /8\right)$ , for all |¡|≤N . Let ${\left\{x_{k}\right\}}_{k\in \mathbb{Z}}$ be a Γ▁ ergodic pseudo trajectory for H , that is $d\left(H\left(x_{k}\right),x_{k+1}\right)<\Gamma$ , for all k except a set of density zero.

Since H is assumed to be a local homeomorphism, this guarantees the existence of compatible local selections, which allows us to construct sequences in the inverse limit space $X_{H}$ ​ that are consistent with the dynamics of H .

We now construct, for each k∈ℤ , a sequence $S^{k}={\left(x_{¡}^{k}\right)}_{¡\in \mathbb{Z}}\in X_{H}$ , such that $x_{0}^{k}=x_{k}$ and $x_{¡+1}^{k}\in H\left(x_{¡}^{k}\;\right)$ for all ¡∈ℤ .

Using the definition of the metric $\tilde{d}$ on $X_{H}$ and the choice of Γ , it follows that $\tilde{d}\left(\sigma_{H}\left(S^{k}\right),S^{k+1}\right)<\tau$ .

for all k except possibly a set of density zero. Hence, ${\left(S^{k}\right)}_{k\in \mathbb{Z}}$ ​ is a τ -ergodic pseudo trajectory of $\sigma_{H}$ .

Since $\sigma_{H}$ ​ has ESP, there exists a point $\tilde{y}={\left(y_{¡}\right)}_{¡\in \mathbb{Z}}\in X_{H}$ . Such that

$$\lim_{\eta \;\to \;\infty }\frac{1}{2\eta +1}\left(\mathrm{card}\left(k\in \left[-\eta ,\eta \right]:\tilde{d}\left(\sigma_{H}^{k}\left(\tilde{y}\right),S^{k}\right)\geq \epsilon \right)\right)=0$$

By the definition of the metric $\tilde{d}$ , this implies in particular that $d\left(y_{k},x_{k}\right)<\epsilon$ , for all k except a set of density zero. Therefore, the sequence $\left\{x_{k}\right\}$ is ϵ▁ ergodically shadowed by the trajectory of $y_{0}$ ​, and hence H also has ESP.■

Theorem 3.4. Let X be a compact metric space and let H be a continuous set-valued mapping which is locally a homeomorphism. If the shift mapping $\sigma_{H}$ ​ on the inverse limit space $X_{H}$ ​ has the ISP, then H also has the ISP.

Proof: Let ϵ>0 . Since $\sigma_{H}$ has the ISP, there exists τ>0 such that for every τ▁ method $\tilde{ⱷ}$ of $\sigma_{H}$ , and every $\tilde{x}\in X_{H}$ ​, there exists $\tilde{y}\in X_{H}$ ​ such that $\tilde{d}\left(\sigma_{H}^{k}\left(\tilde{x}\right),{\tilde{ⱷ}}_{k}\left(\tilde{y}\right)\right)<\epsilon$ , for all k∈ℤ .

Let α=diam(Ⲭ) , and choose N∈ℕ so that $\left(\frac{\alpha }{2^{N-1}}\right)<\left(\frac{\tau }{4}\right)$ . Since H is uniformly continuous, there exists Γ>0 such that d(x,y)<Γ means $d\left(H^{¡}\left(x\right),H^{¡}\left(y\right)\right)<\left(\tau /8\right)$ , for all |¡|≤N . Let $ⱷ:X\to X^{Ζ}$ be a Γ▁ method of H , and take any x∈X .

Since H is a local homeomorphism, this guarantees the existence of compatible local selections, which allows us to lift sequences from X to $X_{H}$ ​. Choose $\tilde{x}=\left(x_{¡}\right)\in X_{H}$ ​ such that $\pi_{0}\left(\tilde{x}\right)=x$ . We define a mapping $\tilde{ⱷ}:X_{H}\;\to \;{X_{H}}^{\mathbb{Z}}$ be lifting ⱷ , in such a way that $\pi_{0}\left(\tilde{{ⱷ}_{k}}\left(\tilde{x}\right)\right)={ⱷ}_{k}\left(x\right),\forall k\in \mathbb{Z}$ . So that $\tilde{ⱷ}$ a τ▁ method for $\sigma_{H}$ .

Since $\sigma_{H}$ ​ has ISP, there exists $\tilde{y}={\left(y_{¡}\right)}_{¡\in \mathbb{Z}}\in X_{H}$ such that $\tilde{d}\left(\sigma_{H}^{k}\left(\tilde{x}\right),{\tilde{ⱷ}}_{k}\left(\tilde{y}\right)\right)<\epsilon$ , for all k∈ℤ . We obtain in particular $d\left(x_{k},{ⱷ}_{k}\left(y\right)\right)<\epsilon$ , where $y=\pi_{0}\left(\tilde{y}\right)$ . Hence, H has the ISP.■

## 4. Conclusion

In this paper, we present a new definition of the inverse and ergodic shadowing properties for set-valued mappings. We explore the relationship between the inverse shadowing property and the ergodic shadowing property of set-valued mappings, as well as their connection to the shift mapping $\sigma_{H}$ on the inverse limit space.

## Data Availability

Our article type does not require data.
