# Supplementary material for: On Ergodic and Inverse Shadowing Properties of Set-Valued Mapping
Source: F1000Res. 2026 Apr 16;15:299. Originally published 2026 Feb 20. [Version 2] doi: 10.12688/f1000research.173380.2 (PMC13096784; doi:10.12688/f1000research.173380.2)
Supplement: Peer review history

# Reviewer report: Reviewer response for version 2

**DOI:** 10.5256/f1000research.198178.r475724

**Published:** 2026-04-21

**Authors:** Abdul Gaffar Khan

**Affiliations:** 1 University of Delhi, Delhi, New Delhi, India

No comments

Is the work clearly and accurately presented and does it cite the current literature?

No

If applicable, is the statistical analysis and its interpretation appropriate?

Not applicable

Are all the source data underlying the results available to ensure full reproducibility?

No source data required

Is the study design appropriate and is the work technically sound?

Partly

Are the conclusions drawn adequately supported by the results?

Yes

Are sufficient details of methods and analysis provided to allow replication by others?

Partly

Reviewer Expertise:

Topological Dynamics

I confirm that I have read this submission and believe that I have an appropriate level of expertise to confirm that it is of an acceptable scientific standard.

# Reviewer report: Reviewer response for version 1

**DOI:** 10.5256/f1000research.191190.r461381

**Published:** 2026-03-13

**Authors:** Syahida Che Dzul-Kifli

**Affiliations:** 1 Universiti Kebangsaan Malaysia, Bangi, Malaysia

1) The abstract should clearly indicate the principal results obtained regarding the relationship between the inverse shadowing property and the ergodic shadowing property for set-valued mappings.

 2) In the Introduction section, several sentences lack clarity and the logical flow between them is weak. The connection between some statements and the main objective of the paper is not clearly established. Furthermore, the discussion of the cited works is rather limited, and their relevance to the present study is not sufficiently explained.

 3) The subsection 1.1 Non–autonomous discrete system mainly introduces terminology and basic concepts. It would be more appropriate to restructure this part as a separate section, e.g., Section 2: Terminology and Basic Concepts (or Preliminaries)

 4) In 1.1, the statement that the mapping is a set-valued mapping on the space may require clarification, since the mapping is actually defined on the hyperspace consisting of compact subsets of the space rather than directly on the space itself.

 5) In Section 1.1, the exposition in this section would benefit from clearer explanation and notation. For example, several concepts are introduced without sufficient description, such as the set of pseudo-trajectories, the index sets used to measure deviations along a sequence, and the mapping that generates sequences of points. The authors should explain these objects more clearly, indicate their roles in the definitions of pseudo-trajectories and inverse shadowing and cite appropriate references for the definitions and terminology introduced in this section.

 6) The Section 2 on results would benefit from a short introductory paragraph summarizing the main results. Moreover, if Section 1.1 is reorganized as a preliminaries section as suggested above, the numbering of the sections should be adjusted accordingly (e.g., the current Section 2 may become Section 3).

 7) The assumptions of the Theorem 2.1 require clarification. In particular, the notions of continuity and surjectivity for the set-valued mapping should be specified, and an appropriate reference for the construction of the inverse limit space in this context would be helpful.

 8) The proof of Theorem 2.1 seems to omit some intermediate steps, particularly in the passage from pseudo-trajectories in the inverse limit space to pseudo-trajectories for the original mapping, and in the final construction of the shadowing sequence. These arguments should be justified more carefully.

 9) Similar comments to those for Theorem 2.1 apply to Theorem 2.2, particularly regarding the clarity of the theorem statement, the density of the notation, and the need for more detailed justification of several steps in the proof. If similar arguments relating shadowing properties and inverse limit spaces appear in the literature, appropriate references should be provided.

 10) Similar comments to those for Theorems 2.1 and 2.2 apply to Theorem 2.3 regarding the clarity of the statement, the notation used in the proof, and the justification of several intermediate steps. In addition, the notion of a local homeomorphism for the set-valued mapping should be clarified.

 11) Similar comments to those for Theorems 2.1–2.3 apply to Theorem 2.4, particularly concerning the clarity of the assumptions, the density of the notation in the proof, and the need for clearer explanation of several intermediate steps.

 12) The novelty and significance of the theorems should be clarified in relation to existing results on shadowing properties and inverse limit spaces. In addition, some assumptions (such as continuity, surjectivity, and the notion of a local homeomorphism for the set-valued mapping) should be more clearly defined or supported by appropriate references.

 13) If the results are correct, they could provide a useful extension of known relationships between shadowing properties and inverse limit spaces to the setting of set-valued dynamical systems. However, the authors should clarify more explicitly how their results differ from or extend the existing theory for single-valued dynamical systems.

 14) The conclusion section is relatively brief and mainly restates the objectives of the paper. It would be helpful if the authors summarize the main results more clearly and emphasize the novelty and significance of the findings, as well as possible directions for future research.

Is the work clearly and accurately presented and does it cite the current literature?

Partly

If applicable, is the statistical analysis and its interpretation appropriate?

Not applicable

Are all the source data underlying the results available to ensure full reproducibility?

No source data required

Is the study design appropriate and is the work technically sound?

Partly

Are the conclusions drawn adequately supported by the results?

Yes

Are sufficient details of methods and analysis provided to allow replication by others?

Partly

Reviewer Expertise:

Dynamical systems, topology, and fuzzy mathematics.

I confirm that I have read this submission and believe that I have an appropriate level of expertise to confirm that it is of an acceptable scientific standard, however I have significant reservations, as outlined above.

# Response

**Published:** 2026-03-27

**Authors:** farah wattan

**Affiliations:** University of Babylon, Iraq

We thank the reviewer for the constructive and helpful comments.

- The abstract and introduction have been improved to clearly reflect the main contributions.
- The structure of the paper has been reorganized for better clarity.
- Definitions and notation have been refined and clarified.
- The proofs have been strengthened with additional details and explanations.
- The assumptions and conditions used in the theorems have been clearly stated and justified.

We believe that the revised manuscript fully addresses the reviewer’s comments and meets the expected scientific standards.

# Reviewer report: Reviewer response for version 1

**DOI:** 10.5256/f1000research.191190.r461379

**Published:** 2026-03-12

**Authors:** Abdul Gaffar Khan

**Affiliations:** 1 University of Delhi, Delhi, New Delhi, India

The results seem to be publishable at first glance, but the quality of the writing and structure needs to be improved a lot. Authors are highly recommended to do the careful reading and address the comments below. Note that these comments are just few examples that needs to be addressed throughout the paper and so after careful reading you might find many more such changes. I am just writing

 1.English sentences and grammar are poorly structured. For eg. "in Introuduction section i.e. [Line 2, Page 3/7 of pdf] 'shadowing approximation trajectories -> shadowing by approximated trajectories", "in section 1.1 According to this mapping sequence . - > fullstop needs to be addressed" etcetera"

 2. Some references are not properly cited for example see Section 1 - Para 2 and Para 3

 3. Section 1 claims that authors are going to provide examples but the paper does not contain any example

 4. Standard sentences and necessary definitions are not even written properly in standard form mathematics. for example 'k(X) is the hyperspace of X" is improper sentence structuring.

 5. Use of non-standard notations should be avoided. For example, use x, y,z etc for elements of $X$ instead of curl letters, do not use greek letters for integeres etc

 5. The paper does not study Non-autonomous systems and thus it is absoulutely absurd to see this section in paper.

 6. The work is done on the induced dynamical systems and not on the set-valued systems. Set-valued systems correspond to a map which takes one element of a space to a subset but the systems author studied takes one element to one element of space. Thus term set-valued should be dropped.

 7. Check definition of density

 8. Definition of ESP is not what it should be. it is written with no care.

 I am skipping a lot of observations that need to be addressed by the authors after reading the paper with extreme care. Only after that it will be easier to decide the quality of the results as in this stage this paper is not reader friendly.

Is the work clearly and accurately presented and does it cite the current literature?

No

If applicable, is the statistical analysis and its interpretation appropriate?

Not applicable

Are all the source data underlying the results available to ensure full reproducibility?

No source data required

Is the study design appropriate and is the work technically sound?

Partly

Are the conclusions drawn adequately supported by the results?

Yes

Are sufficient details of methods and analysis provided to allow replication by others?

Partly

Reviewer Expertise:

Topological Dynamics

I confirm that I have read this submission and believe that I have an appropriate level of expertise to state that I do not consider it to be of an acceptable scientific standard, for reasons outlined above.

# Response

**Published:** 2026-03-27

**Authors:** farah wattan

**Affiliations:** University of Babylon, Iraq

We thank the reviewer for the careful evaluation and valuable remarks.

- The notation has been standardized throughout the manuscript to improve consistency and readability.
- The definition of set-valued mapping has been corrected and clarified as a mapping of the form H: X → K(X).
- The role of the hyperspace K(X) and the Hausdorff metric has been clearly explained.
- The definitions of pseudo-trajectories, δ-methods, and ergodic shadowing have been rewritten with precise mathematical formulation.
- All proofs have been revised to include detailed justifications and to ensure mathematical rigor.

These revisions have significantly improved the clarity and correctness of the manuscript.

# Reviewer report: Reviewer response for version 1

**DOI:** 10.5256/f1000research.191190.r461372

**Published:** 2026-03-09

**Authors:** Khundrakpam Binod Mangang

**Affiliations:** 1 Manipur University, Imphal, Manipur, India

This paper explores the intersection of set-valued dynamical systems and shadowing theory, focusing on the Ergodic Shadowing Property (ESP) and the Inverse Shadowing Property (ISP).

 Recommendation: Accept / Highly Favourable

 1. Theoretical Significance

 The research successfully bridges a gap in contemporary global dynamical systems theory by extending ergodic shadowing concepts to hyperspaces (sets of compact subsets). By proving that the ESP is preserved under the shift mapping of the inverse limit space (Theorem 2.1), the authors provide a vital link between the internal dynamics of a set-valued mapping and its asymptotic topological structure.

 2. Innovation in Definitions

 The paper introduces a rigorous new framework for $\delta$-methods and ergodic pseudo-trajectories within set-valued systems. This is a timely contribution, following the trajectory of recent work by Koo and Lee (2024, 2025), and moves the field forward by shifting the focus from single-point trajectories to set-valued evolutions.

 3. Mathematical Rigour

 The proof for Theorem 2.1 demonstrates a sophisticated use of the Hausdorff metric ($d_H$) and the product topology metric on the inverse limit space. The authors correctly handle the complexities of uniform continuity and density limits, ensuring the results are robust.

 4. Future Potential

 The introduction of a space where elements are themselves sets opens new doors for structuring examples in non-autonomous discrete-time systems. This provides a strong foundation for future research into the stability and "inverse" behavior of complex systems.

 Conclusion:

 This is a well-structured and technically sound contribution to the field. It provides both a theoretical synthesis of existing literature (Román-Flores, Al-Sharaa, etc.) and original results that are essential for researchers studying the stability of discrete dynamical systems.

Is the work clearly and accurately presented and does it cite the current literature?

Yes

If applicable, is the statistical analysis and its interpretation appropriate?

Not applicable

Are all the source data underlying the results available to ensure full reproducibility?

No source data required

Is the study design appropriate and is the work technically sound?

Yes

Are the conclusions drawn adequately supported by the results?

Yes

Are sufficient details of methods and analysis provided to allow replication by others?

Yes

Reviewer Expertise:

Topological Dynamics

I confirm that I have read this submission and believe that I have an appropriate level of expertise to confirm that it is of an acceptable scientific standard.

# Response

**Published:** 2026-03-27

**Authors:** farah wattan

**Affiliations:** University of Babylon, Iraq

We thank the reviewer for the detailed and insightful comments.

- The abstract has been revised to clearly state the main results and highlight the relationship between inverse shadowing and ergodic shadowing properties for set-valued mappings.
- The introduction has been rewritten to improve clarity and logical flow and to better explain the relevance of the cited works to our study.
- The preliminary material has been reorganized into a separate section titled "Preliminaries."
- All definitions and notation have been clarified, especially those related to pseudo-trajectories, methods, and ergodic shadowing.
- The assumptions in all theorems (continuity, surjectivity, and local homeomorphism) are now explicitly stated.
- All proofs have been expanded by adding missing intermediate steps and improving explanations.
- The novelty and contribution of the results have been clarified, emphasizing the extension from single-valued to set-valued dynamical systems.
- The conclusion has been expanded to better summarize the results and highlight their significance.
